# Supplementary material for: FERN – a Java framework for stochastic simulation and evaluation of reaction networks
Source: BMC Bioinformatics. 2008 Aug 29;9:356. doi: 10.1186/1471-2105-9-356 (PMC2553347; doi:10.1186/1471-2105-9-356)
Supplement: Additional file 1 — FERN distribution, Version 1.3. This archive contains the FERN source code and binaries as well as documentation and example models in FernML and SBML. [file 1471-2105-9-356-S1.zip › fern/doc/javadoc/fern/tools/functions/Probability.ReactionProbability.html]

Probability.ReactionProbability


---


|  |  |  |
| --- | --- | --- |
| **Overview** \| **Package** \| **Class** \| **Use** \| **Tree** \| **Deprecated** \| **Index** \| **Help** | |  |
| **PREV CLASS**   NEXT CLASS | **FRAMES**    **NO FRAMES**     **All Classes** |  |
| SUMMARY: NESTED \| FIELD \| CONSTR \| METHOD | DETAIL: FIELD \| CONSTR \| METHOD |  |


---


## fern.tools.functions Class Probability.ReactionProbability

```
java.lang.Object
  fern.tools.functions.Probability.ReactionProbability
```

**All Implemented Interfaces:**: Probability

**Enclosing interface:**: Probability

---

``` public static class Probability.ReactionProbability extends Object implements Probability ```

Implementation for the reaction probability for the autocatalytic network creation.
The probability is calculated by the formula

`factor / max(l1,l2)`

if
```` l1+l2 > oneToLength, else 1.

Author:
:   Florian Erhard

---

Nested Class Summary

Nested classes/interfaces inherited from interface fern.tools.functions.Probability

Probability.Constant, Probability.ReactionProbability


Constructor Summary

Probability.ReactionProbability(double factor,
int oneToLength)


| Method Summary | |
| --- | --- |
| double | getProb(int l1, int l2)             Gets the probability depending on two integers. |

Methods inherited from class java.lang.Object

clone, equals, finalize, getClass, hashCode, notify, notifyAll, toString, wait, wait, wait

Constructor Detail

### Probability.ReactionProbability

```
public Probability.ReactionProbability(double factor,
                                       int oneToLength)
```


Method Detail

### getProb

```
public double getProb(int l1,
                      int l2)
```

:   Description copied from interface: Probability
:   Gets the probability depending on two integers.

    :   Specified by:: getProb in interface Probability
    :   Parameters:: l1 - interger 1: l2 - integer 2 Returns:: probability


---


|  |  |  |
| --- | --- | --- |
| Overview \| Package \| Class \| Use \| Tree \| Deprecated \| Index \| Help | |  |
| PREV CLASS   NEXT CLASS | FRAMES    NO FRAMES     All Classes |  |
| SUMMARY: NESTED \| FIELD \| CONSTR \| METHOD | DETAIL: FIELD \| CONSTR \| METHOD |  |


--- ````
